# Neurasthenia and the Army

**Published:** 1917-06-30

**Authors:** 


The Hospital

The Workers' Newspaper of Administrative Medicine and Institutional Life, Administration, National Insurance and Health.

No. 1621, Vol. LXII. SATURDAY, JUNE 30, 1917. PRICE ONE PENNY

### NEURASTHENIA AND THE ARMY.

Neurasthenia is a term of which much has been heard in recent years. In addition to its strictly medical service it has received the doubtful compliment of a public welcome. Around afternoon-tea tables and in family conferences the word is familiar, and in the Law Courts judges and barristers use it with an easy confidence. To ask for a definition from any of these sources would be a vain thing, and the question would probably be regarded as an index of ignorance. Even the habitual medical expert witness, to whom on various occasions the word is a welcome retreat, appears to prefer it unqualified and undefined. What may be taken as certain is that in such circumstances neurasthenia as applied to its victims has no evil implication. "Malingering," of course, affirms terrible things, and even "hysteria" suggests a measure of discredit, perchance of wantonness, while "functional disease" is too cold an appeal to prompt the flow of human sympathy. But "neurasthenia" has a truly sonorous quality, and clothes those who bear its label with a dignity which invites almost universal respect. There may be here and there a severely critical mind which mocks at all this vagueness and accuses the word as a mere convenient cloak for ignorance. But the general impression holds good and "neurasthenia" continues to command a ready public and to save not a few from the trouble of thinking. For our own part we have no hesitation in saying that, though put to uses of which some are trivial and some ignoble, the word has its clinical value and even necessity, and that employed in a severe and restricted fashion it includes and names actually existing conditions. Oliver Wendell Holmes once wrote of the man who had had "a nerve tapped," and the phrase carries much to the experienced ear.

If, as is commonly and not unreasonably assumed, neurasthenia is provoked by the stress and strain of modern life it is not surprising that the terrors and horrors of the fighting-line have multiplied the number of its victims. So decidedly is this the case that a Special Medical Board has been organised to deal with the sufferers, and new machinery, extending from "neurological centres" immediately behind the fighting-line to "Homes of Recovery" on this side of the Channel, has been brought into operation. We think that this movement merits in its motive a cordial word of recognition. And this all the more that, wide as is the popular sympathy for the civilian neurasthenic, it hardly reaches the neurasthenic soldier. The conjunction of these two terms makes the ordinary man look askance and inclines him to fall back on explanations having an evil import. "Shell-shock" in the warrior is quite in the list of credible and creditable happenings, but neurasthenia in fighting men seems to verge on the effeminate. The Army authorities, however, have to deal with the actual situation, and this does without question include a large number of individuals who, though free from all evidences of organic disease and injury, are yet incapable of military service even in its mildest and most attenuated forms. The practical problem from the military point of view is—What is to be done with these men? The rough-and-ready martinet is inclined to growl that there is nothing the matter with them and that they ought to be sent back to duty under the threat of military law. Possibly the prescription would occasionally succeed, but its general application would be wicked beyond words, and in any event it is impossible. Obviously the way of common-sense is to examine these incapacitated men and to arrange them in classes according to the nature of their maladies and the probable developments. For, without question, not all of them are truly neurasthenic. Some few are deliberate shirkers; others are imperfect convalescents after various acute or sub-acute illnesses; and in a third group must be placed men who are at the commencement of organic nervous disease. When these and other groups are put on one side there will remain the cases which may be justifiably classed as neurasthenics; and the care or cure of these demands a special discipline.

Such, as we gather from a recent lecture by Sir John Collie, is the plan which the Medical Board has actually adopted, and we read with interest of the therapeutic scheme which is being put into operation. The main motive of this scheme, as is the case with all military medical measures, is to get the man back to military service or, failing this, to put him out of the Army as useless. The work thus involves extremely difficult judgments alike in diagnosis and in prognosis, and we shall hope that, in spite of the pressure of the daily task, the Board will be able to give some scientific account of the standards adopted and of the justification or otherwise which the event has provided for these. We quite appreciate an attempt to enlist public sympathy for "Neurosis A and B," but we trust that something more substantial will come out of the special experience which the Board is accumulating. The abnormal conditions with which they are engaged have their parallels in civil life, and will not cease with the war. How best to deal with them is now being studied on a large scale, and guidance for the future ought to be one of the results.

